# The Genus *Metschnikowia* in Enology

**DOI:** 10.3390/microorganisms8071038

**Published:** 2020-07-13

**Authors:** Javier Vicente, Javier Ruiz, Ignacio Belda, Iván Benito-Vázquez, Domingo Marquina, Fernando Calderón, Antonio Santos, Santiago Benito

**Affiliations:** 1 Unit of Microbiology, Department of Genetics, Physiology and Microbiology, Biology Faculty, Complutense University of Madrid, 28040 Madrid, Spain; javievic@ucm.es (J.V.); javiru02@ucm.es (J.R.); ignaciobelda@ucm.es (I.B.); ivbenito@ucm.es (I.B.-V.); dommarq@bio.ucm.es (D.M.); ansantos@ucm.es (A.S.); 2 Department of Chemistry and Food Technology, Polytechnic University of Madrid, Ciudad Universitaria S/N, 28040 Madrid, Spain; fernando.calderon@upm.es

**Keywords:** non-*Saccharomyces*, *Metschnikowia*, *M. pulcherrima*, *M. viticola*, *M. fructicola*, wine flavor

## Abstract

Over the last decade, several non-*Saccharomyces* species have been used as an alternative yeast for producing wines with sensorial properties that are distinctive in comparison to those produced using only *Saccharomyces* *cerevisiae* as the classical inoculum. Among the non-*Saccharomyces* wine yeasts*, Metschnikowia* is one of the most investigated genera due to its widespread occurrence and its impact in winemaking, and it has been found in grapevine phyllospheres, fruit flies, grapes, and wine fermentations as being part of the resident microbiota of wineries and wine-making equipment. The versatility that allows some *Metschnikowia* species to be used for winemaking relies on an ability to grow in combination with other yeast species, such as *S. cerevisiae*, during the first stages of wine fermentation, thereby modulating the synthesis of secondary metabolites during fermentation in order to improve the sensory profile of the wine. *Metschnikowia* exerts a moderate fermentation power, some interesting enzymatic activities involving aromatic and color precursors, and potential antimicrobial activity against spoilage yeasts and fungi, resulting in this yeast being considered an interesting tool for use in the improvement of wine quality. The abovementioned properties have mostly been determined from studies on *Metschnikowia pulcherrima* wine strains. However, *M. fructicola* and *M. viticola* have also recently been studied for winemaking purposes.

## 1. Introduction

In spontaneous wine fermentation, yeast species other than *Saccharomyces cerevisiae*, such as *Hanseniaspora*, *Pichia*, *Torulaspora*, *Lachancea*, or *Metschnikowia* species, govern the initial phase of alcoholic fermentation, thereby influencing the final sensory character of wines, as previously reviewed [1]. The presence of these yeasts is mainly limited to the first half of the alcoholic fermentation process, since they are quickly replaced by *S. cerevisiae*, which has higher fermentative potential.

*S. cerevisiae* starts to predominate when the level of alcohol begins to be a selective factor, as only a few fermentative yeasts, i.e., those showing high ethanol resistance, can cope. However, with the industrial use of active dry yeasts (ADYs) comprising selected *S. cerevisiae* strains as a widespread and useful tool for reducing industrial risks in winemaking, the contribution of these first-phase non-*Saccharomyces* species tend to be nullified in the majority of industrialized wineries.

During the last decade, specific strains of non-*Saccharomyces* species have been reported as being able to improve different technological and sensory parameters of wine (e.g., aroma complexity, color stability, acidity, polysaccharide production, and clarification) [2,3]. Their use seems to be focused on reducing the ethanol content of wines and increasing the release of mannoproteins together with the impact of hydrolytic enzymes in extracting color and aroma precursors from grapes [4,5]. On the contrary, some of the challenges linked with the use of non-*Saccharomyces* species in modern winemaking are related to their low fermentation power and low tolerance to SO_2_, along with difficulties in upscaling production for ADYs. In addition, the use of some non-*Saccharomyces* species has been reported to result in negative consequences for wines for reasons that are related to their sluggish fermentation or excessive production of acetic acid, ethyl acetate, acetaldehyde, or acetoin [6].

Among the non-Saccharomyces yeasts, *Metschnikowia* is a genus of ascomycetous yeasts characterized by cells containing a large oil drop with multilateral budding as well as the formation of large needle-shaped spores in elongated asci. Currently, the number of described *Metschnikowia* species exceeds 80 [7,8], with *M. pulcherrima*, *M. fructicola*, and *M. viticola* most commonly found in wine-related environments [9,10,11].

*M. pulcherrima*, which is ovoidal in shape, usually reproduces by budding, although pseudohyphal growth can be observed under anaerobic conditions. Over the years, *M. pulcherrima* has come to be known by a multitude of different names due to changes in its classification based on classical methods on yeast taxonomy (*Asporomyces uvae*, *Candida pulcherrima*, *Castellania castellanii*, *Cryptococcus castellanii*, *Eutorula pulcherrima*, *Monilia castellanii*, *Rhodotorula pulcherrima*, *Saccharomyces pulcherrimus*, *Torula pulcherrima*, *Torulopsis pulcherrima*, *Torulopsis burgeffiana*, *Torulopsis dattila*, etc.) [8]. 

*M. pulcherrima* shows a low to moderate fermentative power, as is the case with most non-*Saccharomyces* species that are present in grapes. This species is not able to tolerate ethanol concentrations over 4–5% (*v*/*v*), so it naturally disappears during alcoholic fermentation when this concentration of ethanol is exceeded. Furthermore, although *M. pulcherrima* shows moderate resistance to SO_2_, higher than for some other non-*Saccharomyces* wine species, this tolerance is always lower than that observed for *Saccharomyces* wine strains [12,13]. For these reasons, *M. pulcherrima* should be used in mixed fermentations with a yeast that exhibits a higher fermentative power, such as *S. cerevisiae*, which will then be the species responsible for finishing alcoholic fermentation [14]. Accordingly, dehydrated yeast manufacturers recommend the use of selected *M. pulcherrima* strains in sequential inoculation with *S. cerevisiae* in order to maximize its effects on determined characteristics in the final product, as is usually done with other commercial non-*Saccharomyces* species such as *T. delbrueckii*, *L. thermotolerans*, or *Pichia kluyveri* [15]. 

In this review, the interesting antimicrobial properties of *M. pulcherrima* are also considered in the context of winemaking. It has been reported that *M. pulcherrima* shows strong biocontrol activity against various yeast genera that are considered detrimental in enology (*Brettanomyces/Dekkera*, *Hanseniaspora*, and *Pichia*) and several filamentous fungi that cause grape spoilage (*Penicillium* sp., *Aspergillus* sp., and *Fusarium* sp.), while having a low to null effect on *S. cerevisiae* performance. The production of the iron sequestering brown-red pigment pulcherrimin is responsible for this observed inhibition [16,17]. In addition, the presence of killer toxins cannot be ruled out as their production has been described for other *Metschnikowia* species [18].

Although it is widely assumed that *M. pulcherrima* may exert a positive impact in many wine quality parameters, several authors have reported significant strain-dependent variability [19]. This review discusses properties that have already been studied in different strains of *M. pulcherrima* that may positively impact wine quality [20]. Additionally, we discuss some key operational concepts for enhancing positive attributes through the use of an *M. pulcherrima* inoculum in wine fermentation.

In the last decade, *M. pulcherrima* has been studied in detail in terms of its relation to the production of wine [21]. However, other species of the same genus, such as *M. viticola* [22] and *M. fructicola* [23], are also receiving some scientific attention, and development of their application within the wine industry has already begun.

It is well known that non-*Saccharomyces* yeasts are responsible for the production of a great variety of enzymes that contribute to the organoleptic characteristics of wines. Among them, the *Metschnikowia* genus stands out because of its ability to produce hydrolytic enzymes (glycosidases, proteases, and pectinases), which may directly impact the sensorial and technological properties of wine [24].

In summary, the *Metschnikowia* genus possesses different characteristics that could be utilized in different industrial or agricultural processes. Taking advantage of those characteristics, different strains have been selected and commercialized for different uses (Table 1).

Thus, the aim of the present work is to compile and synthetize the results and conclusions obtained from different research groups during the last few years. To facilitate understanding and management, different enological traits of *M. pulcherrima* have been summarized (Table 2).

## 2. The Genus *Metschnikowia*: Scientific and Enological Contexts

According to the NCBI taxonomy browser, the family *Metschnikowiaceae* currently comprises five different genera, namely, *Aciculoconidium*, *Clavispora*, *Kodamaea*, *Metschnikowia*, and *Nectaromyces*. The genus *Metschnikowia* has been defined as a clade containing 81 different species [30,31]. Most *Metschnikowia* species are not ecologically widespread, but they show a high degree of specialization [32,33]. Members of the *M. pulcherrima* clade appear to be primarily associated with fruit-feeding insects. In vineyard- and wine-related environments, the species *M. pulcherrima, M. fructicola*, and *M. viticola* have been reported [10]. In this work, *M. viticola* was apparently easy to differentiate from *M. pulcherrima* and *M. fructicola* from both a phylogenetic and phenotypic point of view. However, *M. pulcherrima* and *M. fructicola* are difficult to separate when examining wine-related traits or by sequencing generally accepted ribosomal DNA markers (ITS or 26S rDNA partial sequencing) [34]. Fortunately, they can be differentiated by comparing the nucleotide sequence of the EF2 (elongation factor 2) marker [32], which shows around 9% sequence divergence between these species.

**Figure 1:**
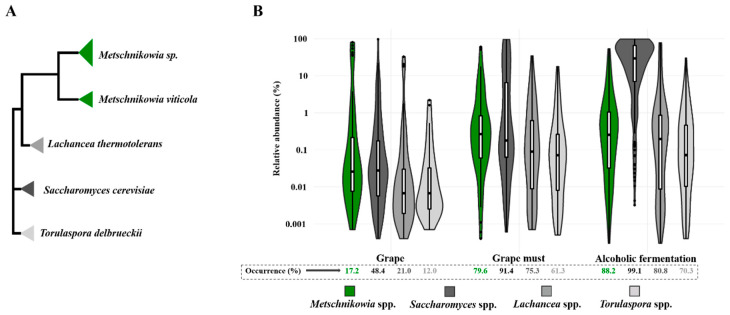
(**A**) Scheme of the phylogenetic relationship between Metschnikowia sp. (including isolates of *M. pulcherrima* and *M. fructicol*a species), *M. viticola, Saccharomyces cerevisiae, Torulaspora delbrueckii*, and *Lachancea thermotolerans*, using 26S rRNA sequences. (**B**) Occurrence rates and relative abundance values at different winemaking stages for the above-mentioned yeast genera (personal communication from A. Acedo; values estimated from a large-scale NGS amplicon-based survey).

Figure 1A shows a scheme of the phylogenetic relationship (based on 26S rDNA sequences) between the *Metschnikowia* species, *S*. *cerevisiae*, and two of the main other non-*Saccharomyces* yeasts commonly commercialized in wine industry (*L. thermotolerans* and *T. delbrueckii*). As can be deduced from their functional traits (fermentation capacity, ethanol, and SO_2_ tolerance), *T. delbrueckii* is closely related with *S. cerevisiae* in comparison with the *L. thermotolerans* and *Metschnikowia* yeasts, which contrarily show a notably a lower fermentation capacity and lower ethanol and SO_2_ tolerance than the *S. cerevisiae* and *T. delbrueckii* species. However, Figure 1B shows that the *Metschnikowia* and *Lachancea* species have a higher occurrence rate than the *Torulaspora* species in grape and wine samples. In fact, the *Metschnikowia* species appeared in grape and grape must samples with abundance patterns like those of *Saccharomyces* species.

**Figure 2:**
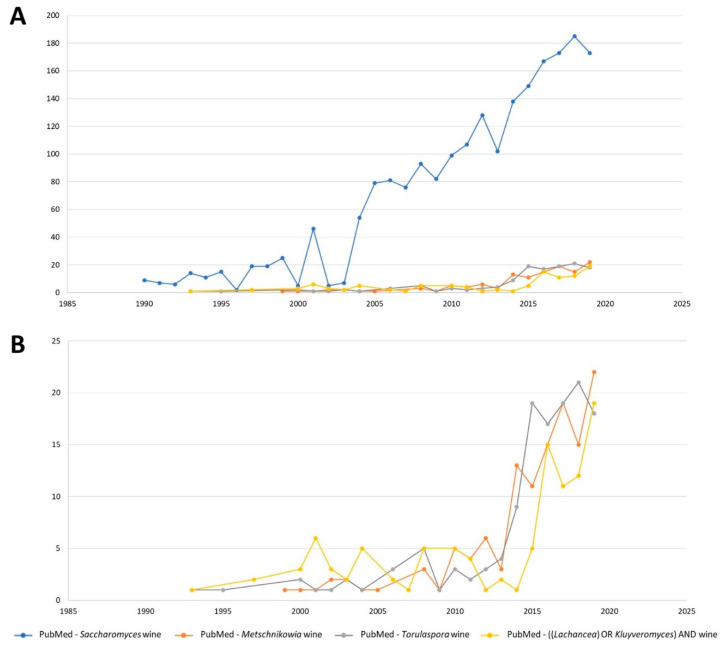
Graphical representation of the number of publications by year (1990 to 2020) related to a yeast genus and wine search in PubMed. (**A**) Results looking for *Saccharomyces*, *Metschnikowia*, *Torulaspora*, and *Lachancea* (or *Kluyveromyces*). (**B**) Detail of the results looking for *Metschnikowia*, *Torulaspora*, and *Lachancea* (or *Kluyveromyces*).

In examining the scientific interest in the abovementioned wine-relevant non-*Saccharomyces* species (using a PubMed^®^ search with the following search strategy: “(Genus_name) AND wine”, we can highlight that in terms of wine-related studies, the first mention of *Torulaspora* yeasts was in 1973 (though such reports only started to appear regularly since 1987), with reports of Metschnikowia appearing in 1999 and, more recently, *Lachancea* (including searches for *Kluyveromyces thermotolerans*, by which the currently named *L. thermotolerans* was formerly known) in 2001 (Figure 2). However, in 2019, *Metschnikowia* was mentioned in 22 wine-related scientific works and the *Torulaspora* and *Lachancea* yeasts in 19 and 18 works, respectively. Thus, in last few years, the increasing incidence of Metschnikowi*a* yeasts occurring in wine-related environments and their increasing number of potential uses in improving wine quality mirror the greater attention from researchers. 

The complete genome sequences of different strains of *M. pulcherrima* and *M. fructicola*, isolated from different environments (soil, insects, grapes, etc.), are already available [35,36,37,38] and are useful in guiding future research efforts on the basic and applied aspects of *Metschnikowia*. These resources open new possibilities for understanding the transcriptional regulation of the various *Metschnikowia* species [35] as a necessary step for their wider and more controlled use in industrial and in-field operations.

## 3. Fermentation Parameters

### 3.1. Fermentation Capacity 

Ethanol production under winemaking conditions is directly linked to the fermentation capacity of the utilized yeast and its tolerance to ethanol, which is low in the case of *Metschnikowia*. Some strategies for ethanol reduction in wine are based on using non-*Saccharomyces* yeasts, due to their low fermentative yield, or due to the possibility of forcing a Crabtree-negative effect, thereby consuming sugars by respiration [39]. The remaining sugar that it is not converted into ethanol may be transformed into other compounds, such as glycerol or acids [26]. Nevertheless, in some cases, the residual sugar present at the end of the fermentative process is extremely high (up to 150 g/L) [40].

The fermentative capacity of *M. pulcherrima* is limited under single-inoculation fermentation conditions. Most studies have reported *M. pulcherrima* as producing up to 4.5% (*v*/*v*) ethanol [41]. This moderate fermentation power makes it impossible to use a single *Metschnikowia* inoculum for wine fermentation, or even for lower ethanol beverages such as beer. However, some strains of *M. pulcherrima* are able to produce around 9% (*v*/*v*) ethanol, though this process takes 180 days and also consumes almost all the sugar [42]. This is the reason why *M. pulcherrima* is preferred for improving quality parameters such as the aroma in sequential fermentations where it is combined with more fermentative yeasts such as *S. cerevisiae*. 

Despite its resistance to all *S. cerevisiae* killer toxins and the absence of any antagonistic interactions between *M. pulcherrima* and *S. cerevisiae* [40,43]; when inoculated together at equal cell density, the *M. pulcherrima* population decreases rapidly due to its low ethanol resistance [26]. The use of sequential inoculation strategies could solve this problem derived from the low ethanol resistance of *M. pulcherrima.*

The effect of the sequential inoculation depends on the initial density of the *S. cerevisiae* population used in the second inoculation [19], the specific strains that are used, and the combinations [44]. Several studies have shown slight differences, with an observed decrease in ethanol production ranging from 1.39% to 0.2% (*v*/*v*) compared to regular fermentations with only *S. cerevisiae* [19,26,41,45,46]. The use of the sequential fermentation modality usually delays the alcoholic fermentation time by around four days in comparison to the *S. cerevisiae* controls [47].

The fermentation power of *M. pulcherrima* is strain- and culture condition-dependent. The results obtained by some research groups regarding the initial must composition, pH, diammonium phosphate, sulfur dioxide, and temperature indicate the importance of performing studies in order to optimize the parameters that have a major influence in the metabolism of *M. pulcherrima* [40,43].

In addition to *M. pulcherrima*, some other species of *Metschnikowia* have been studied in fermentation. Wines fermented with *S. cerevisiae* in combination with *M. viticola* (and even *M. fructicola)* usually have residual sugar and low ethanol content (10–11.9% ethanol) [22].

### 3.2. Glycerol

Glycerol plays an important role in wine properties because it is associated with different characteristics, such as flavor persistence and oiliness [48]. The use of *Metschnikowia* increases the final concentration of glycerol in wines. Recent works have revealed that the increase in glycerol during sequential inoculation ranges from 4 to 20% [26,29], or even up to more than 40% [41]. The presence of competitors may influence glycerol production, which is higher when the cellular concentration of *S. cerevisiae* is lower [19].

The physicochemical conditions are also decisive in the production of glycerol. The addition of 60 mg/L sulfur dioxide to must decreased the production of glycerol by one-third [43], which could be explained by the low resistance to the oxide [19]. 

### 3.3. Nitrogen Metabolism

The consumption of different nitrogen assimilable sources (NAS) by *M. pulcherrima* was assayed under in vitro conditions. These studies, which examined both single and mixed fermentations employing *M. pulcherrima* and *S. cerevisiae*, reported that at the end of the fermentation a significant amount (up to 40%) of the studied NAS had not been consumed when single cultures of the non-S*accharomyces* species were used, whereas *Saccharomyces* consumed all the nitrogen sources present in the media, including in mixed cultures [49]. In a recent study, *M. pulcherrima* is the least influenced yeast species by the must nitrogen composition [50].

During sequential fermentation with *M. pulcherrima* and *S. cerevisiae*, adequate management of yeast nutrition is essential, especially regarding the nitrogen source. The addition of nitrogen at the moment of *S. cerevisiae* inoculation is recommended for facilitating the completion of the fermentative process [51]. *M. pucherrima* sequential fermentations seems to increase the final amino acid concentration in wine. The higher increments of amino acids are because of increases in isoleucine, serine, and phenylalanine. Those amino acids doubled its concentration compared to the *S. cerevisiae* control. Threonine was the only amino acid that decreased [26].

The use of *M. pulcherrima* in aging on lees also triggers an increase in the final amino acid concentration of wines. During aging, the use of *M. pulcherrima* lees in combination with *S. cerevisiae* increases the final concentration of amino acids compared with the single use of *S. cerevisiae*, especially in the amount of histidine and tryptophan (63%). On the contrary, threonine is highlighted because it is the only amino acid that was observed to decrease [52]. 

### 3.4. Total Acidity 

The inclusion of *Metschnikowia* in must fermentation seems to be associated with slight losses in the total acidity of the final wine as reflected by an increase in pH. The loss in total acidity that affects pH may involve microbial, protein, or color instability.

Variable results have been encountered when considering single *S. cerevisiae* fermentations compared to sequential fermentations using *M. pulcherrima* and *S. cerevisiae*, where the reduction in total acidity in sequential fermentations has been observed to be both statistically nonsignificant (e.g., from pH 3.39 to 3.40) [26,27] and significant (e.g., from pH 3.40 to 4.20) [19,29]. Nevertheless, in some cases, higher final levels in titratable acidity (1.68 g/L) were observed in comparison to the *S. cerevisiae* control [53].

The ratio of tartaric to malic acids will determine the acidity of the wine. Data regarding the influence of *Metschnikowia* on the final tartaric acid concentration are limited, though it seems to be slightly lower (up to 6%) in co-inoculated fermentations [29]. Malic acid is susceptible to being consumed by some non-*Saccharomyces* wine-related species, with *M. pulcherrima* presenting the capacity to degrade it under single and sequential fermentation conditions by around 10% [28,42]. Nevertheless, in some cases, the malic acid content under sequential inoculation may increase [54].

If the same strain of *M. pulcherrima* is used along with two different strains of *S. cerevisiae,* a minimum increase in lactic and citric acid is observed, notwithstanding that they are not statistically significant [21]. That may confirm that lactic and citric acids seem to not be affected by *M. pulcherrima* [26,27,29].

Recent studies have reported increases in fumaric acid for sequential fermentations between *M. pulcherrima* and *S. cerevisiae*, from 30% to 69% (up to 2.9 g/L). The same study reports increases in fumaric acid of up to 66%, depending on the used *M. pulcherrima* strain [41].

### 3.5. Volatile Acidity

According to several works, the decrease in volatile acidity when *M. pulcherrima* is used varies between 10% and 75% [40,41,50], indicating that the reduction of volatile acidity in such conditions seems to be a trend. However, other studies have reported equal quantities of acetic acid when sequential fermentation was employed [21,26], or even an increase of around 20% [29]. In vitro assays have shown that when synthetic must is used that different results are obtained when compared with natural white must. 

### 3.6. Aroma Compounds 

#### 3.6.1. Fermentative Aromas

Higher alcohols show the most irregular results of all the reported enological parameters for *M. pulcherrima*. Some studies show increments of up to 33% in the total concentration of higher alcohols for sequential fermentation involving *M. pulcherrima* compared to the *S. cerevisiae* controls [27,28,53]. However, other studies state the contrary effect, with reductions of around 30% [19,21,26,54]. 2-phenylethanol is always described to increase about 30% when *M. pulcherrima* ferments in sequential fermentations [29,47,53]. Other compounds that increase under sequential fermentation are methionol [28], isoamyl alcohol [53], hexanol [47], and isobutanol [27]. On the other hand, those that generally decrease are 3-methyl-1-butanol [27], hexanol, and benzylic alcohol [26]. The discrepancy observed among the different studies is explained by the high variability observed in higher alcohols metabolism between *M. pulcherrima* strains, which is quantified to be about 29% (1-butanol), 66% (2-phenylethanol), or 65% (1-hexanol) depending on the studied higher alcohol [55].

*M. pulcherrima* usually increases the total concentration of esters [40,47,53]. Shikimic acid is a precursor for different esters such as benzaldehyde or ethyl cinnamate. The final concentration of shikimic acid decreases when *M. pulcherrima* ferments in mixed fermentations together with *S. cerevisiae*. Some studies observed a statically significant loss of shikimic acid corresponding to around 0.66%. This fact could be related with the formation of aromatic compounds from shikimic acid, which could result in a more complex final product [29]. Among all ethyl esters, ethyl octanoate is the most relevant as its concentration increases around 14% [29]. Acetates may decrease (around 30% less) when no-*Saccharomyces* species ferment [26,29,53].

The final total concentration of fatty acids generally increases from 0.52% to 7.5% when *M. pulcherrima* ferments [21,26,29]. Fatty acids such as octanoic or decanoic acids show great variability between studies. However, others such as hexanoic or pentanoic acid always increase when *M. pulcherrima* ferments [21,26,29]. 

Acetoin and diacetyl generally increase when using *M. pulcherrima*. Acetoin increases varied from 10 to 400%, while diacetyl doubled when *M. pulcherrima* fermented [28].

Acetaldehyde usually decreases when non-*Saccharomyces* species ferment, improving the aromatic profile in wine [56]. Some studies report slight differences in acetaldehyde with no statistical significance [19,21,28], while others report statistically significant decreases down to 40% [26] for fermentations involving *M. pulcherrima*.

#### 3.6.2. Varietal Aromas

Volatile sulfur molecules are important aroma compounds, including both pleasant and unpleasant aromas in wine. β-lyase is responsible for the main enzymatic activity involved in varietal thiols released from their S-conjugated nonvolatile precursors. Using non-*Saccharomyces* yeasts is a novel approach to enhancing the thiol profiles of wine [57]. Some *M. pulcherrima* strains present β-lyase activity [58]. *M. viticola* is the *Metschnikowia* species with higher β-lyase activity as 60% of the isolates show the enzymatic activity, while other *Metschnikowia* species show about 10% [10]. 

One study reports thiols concentration (4 methyl-4-sulfanylpentan-2-one (4-MSP) and 3-sulfanylhexan-1-ol (3-SH)) to decrease when *M. pulcherrima* is used with *S. cerevisiae* in mixed fermentations [47]. On the contrary, another study [21] shows a *M. pulcherrima* strain in sequential inoculation with *S. cerevisiae* to increase 4-MSP concentration when compared with *S. cerevisiae* control. The thiol character (i.e., tropical, citrus aromas) of these wines increased as a decrease in the total concentration of higher alcohols, which overshadow the wine bouquet, accompanied thiol production. Most strains of *Metschnikowia* genus do not show sulfite reductase activity. This activity produces H_2_S off-flavor [10,59,60]. A selected *M. pulcherrima* strain with high cystathionine-β-lyase activity released seven times more 4-methyl-4-sulfanylpentan-2-one (4-MSP) than the *S. cerevisiae* control. That strain did not influence the final concentration of acetylated 3-sulphanyl-hexanol (3-SHA) and 3-SH suffered a slight decrement [21]. Another study found traces of the methylated form of methanethiol (MeSH) in all fermentations involving *M. pulcherrima* [29].

Terpenes are the most important varietal aroma compounds in wine. The hydrolytic activity of glycosidase enzymes (mainly β-glucosidase, β-d-xylosidase, and α-l-arabinofuranosidase) release terpenes from their glycosidic nonvolatile precursors. β-Glucosidase activity is not very common among *M. pulcherrima* isolates, being only present in around 25% of the strains [61]. A newer wide survey including different *Metschnikowia* species (*M. viticola, M. pulcherrima*, and *M. fructicola*) reported that most showed β-glucosidase, α-l-arabinofuranosidase, and β-d-xylosidase activities [10]. The commercial strain of *M. pulcherrima* Flavia MP346 increases volatile terpene concentrations in wine because of its extracellular α-l-arabinofuranosidase. One study reports *M. fructicola* to have a positive effect on terpene release during wine fermentation [23]. Although *Metschnikowia* β-glucosidase activity is effective at increasing the level of monoterpenols in wine, some environmental parameters, such as pH, glucose, ethanol, and SO_2_, should be taken into consideration to optimize the release of these desirable aromatic compounds [62]. Contrarily, other works do not report any influence of *M. pulcherrima* on the total terpene concentration under wine fermentation conditions compared to *S. cerevisiae* single fermentation [26]. Several studies reported an increase in the total terpene concentration at the end of sequential fermentations involving *M. pulcherrima* compared to the single fermentation of *S. cerevisiae* up to 100%. Linalool was the most affected by *M. pulcherrima* in the fermentative process [29,47,53]. Regarding β-damascenone, one study reported an increased concentration when *M. pulcherrima* fermented together with *S. cerevisiae* [46,47].

Other *Metschnikowia* species also positively influence wine aroma. A recent study tested the aromatic profile of wines inoculated with autochthonous strains of *S. cerevisiae* combined with *M. fructicola*. The results show that mixed fermentations with *M. fructicola* improved the aromatic complexity of wines, increasing the ester and terpene contents [23]. Using *M. viticola* in sequential fermentation with *S. cerevisiae* increased berry and fruity flavors in the produced wines [22]. The study compared *M. viticola* to other non-*Saccharomyces* species isolated in Denmark, such as *Hanseniaspora uvarum* in sequential fermentations with *S. cerevisiae*.

### 3.7. Polysaccharides and Mannoproteins

Some studies report most *M. pulcherrima* strains to release more polysaccharides than *S. cerevisiae* controls during single alcoholic fermentations with both species [19]. The *M. pulcherrima* strain that performed the best released 17% more polysaccharides than the best *S. cerevisiae* releaser. The same author observed a similar effect for mixed fermentations between *M. pulcherrima* and *S. cerevisiae*. The final polysaccharide concentration increased when the *M. pulcherrima* inoculum was higher. The increases varied from 20 to 50% depending on the *M. pulcherrima* initial inoculum proportion. Other study report *M. pulcherrima* to release higher amounts of mannoproteins than two *S. cerevisiae* controls in 50 and 90%. Nevertheless, another *S: cerevisiae* strain released 37% more mannoproteins than the selected *M. pulcherrima* strain [52].

Ninety percentage of *Metschnikowia* species strains produce protease and pectinase [10]. However, in the case of *M. pulcherrima* some studies report not to be proteolytic [19], while other studies report all studied *M. pulcherrima* strains to possess this enzymatic activity [42]. Protease activity may solve protein haze formation in wines, being an alternative or a complement to the use of bentonite [63]. The role and expression of the protease MpApr1 produced by a *M. pulcherrima* strain have been studied [63,64]. Pectinase activity is strain-dependent on *M. pulcherrima* [65]. 

### 3.8. Color, Anthocyanins, and Polyphenols

Sequential fermentations involving *M. pulcherrima* and *S. cerevisiae* result in wines with a significant reduction in the final concentrations of grape anthocyanins such as delphinidin-3-*O*-glucoside (44%), cyanidin-3-*O*-glucoside (39%), petunidin-3-*O*-glucoside (16%), peonidin-3-*O*-glucoside (29%), and malvidin-3-*O*-glucoside (11%) [27]. This phenomenon is explained because of a higher anthocyanin adsorption by the cell walls of the *Metschnikowia* species or because of the specific adsorption properties of the studied strain. This property is highly strain-dependent on other species such as *S. cerevisiae* [66]. Other studies report the final concentration of vitisins to be 17% lower than the *S. cerevisiae* control [27]. This is explained because of the lower acetaldehyde production of *M. pulcherrima* than the *S. cerevisiae* control of about 27% [26]. Vitisin B integrates acetaldehyde in its molecule and higher acetaldehyde concentrations produce higher levels in vitisin B [66]. *M. pulcherrima* is not a high producer of pyruvic acid. This explains the low formation of vitisin A in fermentations involving *M. pulcherrima* as pyruvic acid is part of Vitisin A molecule [67,68]. Fermentations involving *M. pulcherrima* show lower concentrations in acetylated anthocyanins such as vitisin A-(6’’-acetylglucoside), petunidin-3-*O*-(6’’-acetylglucoside), peonidin-3-*O*-(6’’-acetylglucoside), and malvidin-3-*O*-(6’’-acetylglucoside) by 30%, 10%, 5%, and 10%, respectively. 

The opposite effect takes place for cumarilated anthocyanins, where fermentations involving *M. pulcherrima* show higher concentrations of petunidin-3-*O*-(6’’-*p*-coumaroylglucoside) and malvidin-3-*O*-(6’’-*p*-coumaroylglucoside) by 18% and 22%, respectively. Fermentations involving *M. pulcherrima* co-inoculated with lactic bacteria showed higher final concentrations of *p*-coumaric acid than the *S. cerevisiae* controls by about 25% [69]. Other non-*Saccharomyces* species also produce wines with higher *p*-coumaric acid contents [70,71,72,73]. This explains the higher concentrations of cumarilated anthocyanins. However, some authors recommend selecting strains that release moderate amounts of *p*-coumaric acid, as this compound can evolve into undesirable 4-ethylphenol if contamination by *Brettanomyces/Dekkera* genera takes place [74]. Fermentations involving *M. pulcherrima* show no presence of vinylphenol adducts such as malvidin-3-4-vinylguaiacol, malvidin-3-(600-acetylglucoside)-4-vinyphenol, or malvidin-3-(600-p-coumaroylglucoside)-4-vinylphenol. The *S. cerevisiae* control produced these highly stable color compounds. Other non-*Saccharomyces* species possess higher hydroxycinnamate decarboxylase activity than *S. cerevisiae*, which allows the production of higher concentrations of vinylphenol adducts [71,72]. No studies report this effect for *M. pulcherrima*. One study reported a final color intensity of about 20% lower than the *S. cerevisiae* control for fermentations involving *M. pulcherrima*. This effect took place due to the lower concentrations of most anthocyanins [27]. Nevertheless, co-inoculations of *M. pulcherrima* with lactic bacteria and *S. cerevisiae* showed higher concentrations in total phenolics and malvidin-3-*O*-glucoside than the combined *S. cerevisiae* and lactic acid bacteria control by about 8% and 20%, respectively [69].

One study reports that co-fermentations with *M. pulcherrima* using Shiraz grapes reduced 10% of the tannin content compared to the *S. cerevisiae* controls [75]. Although these fermentations did not show differences in anthocyanin concentrations between treatments including *M. pulcherrima* and those performed with *S. cerevisiae* alone, fermentations involving *M. pulcherrima* showed higher color in about 6%. Co-fermentations involving *M. pulcherrima* showed higher contents of nonbleachable pigments in about 10% [75].

*M. pulcherrima* possess polygalacturonase activity. This activity increases the concentration of anthocyanin and polyphenol compounds while decreasing wine turbidity [5]. 

## 4. Sensory Influence in Wine

There are multiple studies detailing the influence of the *Metschnikowia* species on the organoleptic properties of wines. A sensory evaluation of Muscat of Alexandria wines co-fermented with *M. pulcherrima* showed higher fruity and floral aromas in sequential fermentations than with the *S. cerevisiae* controls [53]. These results were supported, from a chemical point of view, by the higher observed concentrations of molecules such as terpenols, higher alcohols, and esters. One of the aromas that have been observed to be increased when *M. pulcherrima* is used is the “pear character”, which is highly related to higher final contents in ethyl octanoate [24].

Wines obtained with sequential *M. pulcherrima* fermentation exhibit a higher overall impression than the *S. cerevisiae* controls due to the better aroma quality reported for such wines [26]. *M. pulcherrima* fermentations show a higher score in their fruity character, justified due to a lower final concentration of higher alcohols and a higher concentration of specific fruity esters such as ethyl hexanoate, ethyl octanoate, and ethyl decanoate. Additionally, fermentations involving *M. pulcherrima* showed lower oxidative perception, attributed to a lower concentration of ethanal. Fermentations involving *M. pulcherrima* showed a higher Riesling typicity and higher score in Riesling descriptors such as peach/apricot and especially in citrus/grape/fruit character. Further studies correlated higher scores in Verdejo typicity with higher concentrations in thiols in fermentations involving *M. pulcherrima*, especially for thiol 4-methyl-4-sulfanylpentan-2-one, whose content was 5 times higher than the *S. cerevisiae* control [21]. Those results justify the increase in Riesling typicity reported earlier [26], as some of the aroma sensory descriptors are related to thiol compounds [57].

Some studies report fermentations involving *M. pulcherrima* to show lower scores in sensory wine descriptors such as body, astringency, or bitterness than the *S. cerevisiae* controls [27]. The observed lower final tannin concentration justifies those results from a chemical point of view. A study regarding co-inoculated fermentations involving *M. pulcherrima*, *S. cerevisiae*, and lactic acid bacteria showed that panelists preferred this option to the control involving co-inoculation between *S. cerevisiae* and lactic acid bacteria [69]. In the study, fermentations involving *M. pulcherrima* scored lower in astringency and bitterness. However, other authors reported fermentations involving *M. pulcherrima* to show higher astringency than the *S. cerevisiae* control [75].

## 5. Biocontrol Activity

Some researchers have demonstrated that *M. pulcherrima* inhibits the development of *Botrytis cinerea* and other filamentous fungi, yeasts, and bacteria that are undesirable to the wine industry [16]. This inhibition takes place due to the chelation of iron, which inhibits the survival of other species [73]. *M. pulcherrima* also uses this strategy as a main tool for competition, due to the fact that iron is essential for fungal growth and pathogenesis [35]. This may negatively affect the fermentative process when mixed inoculums of *S. cerevisiae* and *M. pulcherrima* are used, due to the amensalism of *M. pulcherrima* to *S. cerevisiae* through iron depletion [49]. That ability has been successfully used in apple fruit trees for the biocontrol of *B. cinerea* [76]. Some authors [77] have demonstrated that in the culture media in which *M. pulcherrima* has been grown for 6 days, there is a 40% reduction in the grey mold McKinney index, and it was shown to be an effective biocontrol tool for controlling the postharvest decay of strawberries. A recent study characterized the genomic regulation of pulcherriminic acid, a pigment that exhibits strong antifungal activity against rhizospheric phytopathogenic fungi [78].

Due to the prohibition of some effective fungicides against *B. cinerea* and other undesirable fungi in the periods before harvesting, the use of biocontrol agents, such as *M. pulcherrima*, could be useful candidates for application in the winemaking industry. *M. pulcherrima* can not only be used for fungal inhibition, but also for the control and reduction of their toxic products, such as ochratoxin A (OTA). This yeast has the ability to reduce OTA by up to 80% at 30 °C after 15 days, with practically no adsorption to the yeast cell wall [79,80].

*M. fructicola*, as well as *M.pulcherrima,* is used as biocontrol tool against filamentous fungi as well as against the spoilage of yeasts. The most important biocontrol molecule in *M. fructicola* is pulcherrimin; nevertheless, its biosynthesis has not yet been characterized [36]. The biocontrol activity of pulcherrimin has been demonstrated against *B. cinerea, Alternaria alternata*, and *Penicillium expansum*. Pulcherrimin is not the only antimicrobial defense mechanism, it also has been demonstrated that among the pull of different gene clusters involved in secondary metabolite production there are two genes involved in the synthesis of terpenes that can be potentially bioactive molecules against *Penicillium digitatum* [36]. 

## 6. Main Selection Criteria for *M. pulcherrima* Strains

The studies that compare fermentations involving different strains of *M. pulcherrima* species report a high strain variability for several wine quality parameters (Table 3). Because of this, some selection criteria should be established to improve the wine quality parameters reported in this review.

**Figure 3:**
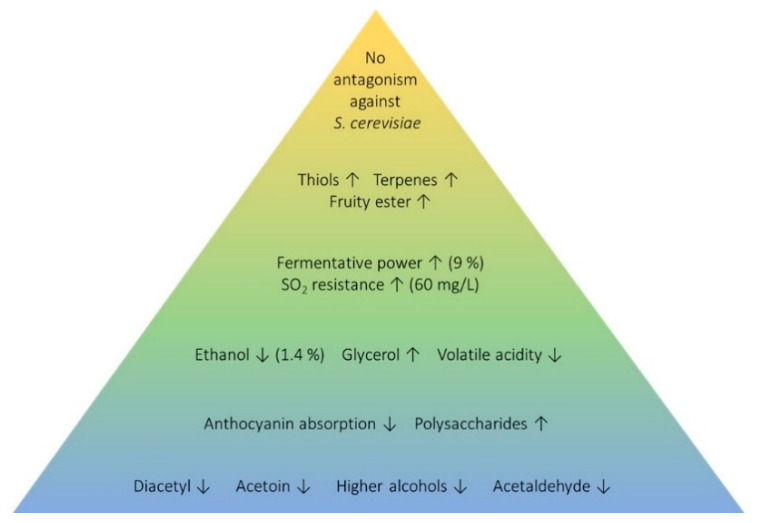
Summary of proposed *Metschnikowia pulcherrima* selection parameters.

In our opinion, the first parameter to take into account in a *M. pulcherrima* selection process must be the absence of antagonism effects against *S. cerevisiae* as any positive effect over wine would have no sense if a proper alcoholic fermentation cannot take place. The main advantage about using *M. pulcherrima* is due to its positive influence on wine aroma. Regarding this fact, *M. pulcherrima* strains should be selected in order to release varietal aroma compounds, such as thiols and terpenes, while producing fruity esters such as ethyl octanoate. Fermentative power together with ethanol and sulfur dioxide resistance should be considered in order to allow the selected strains to perform longer during industrial alcoholic fermentations to make it easy to achieve the desired quality objectives. These parameters remain as the main limitation of the available commercial strains. Other secondary positive effects that could be selected are the ability to reduce the final ethanol content and to increase the glycerol concentration in final wines. Some parameters of additional interest are the anthocyanin absorption and polysaccharides release. Finally, some negative parameters reported for some *M. pulcherrima* strains such as diacetyl, acetoin, higher alcohols, or acetaldehyde must be avoided in the selected strains. Figure 3 summarizes the proposed *M. pulcherrima* selection parameters.

## 7. Conclusions

*M. pulcherrima* has distinct characteristics that make this yeast useful in winemaking in comparison to other non-*Saccharomyces* species of enological interest. Nevertheless, the use of *M. pulcherrima* in enology is not a common option nowadays, but the range of potential applications of this species are vast. Some of the studied parameters are variable, depending on the used strain and the fermentation conditions. This is the reason why more studies should be carried out concerning the use of this yeast in enology. Other new *Metschnikowia* species, such as *M. fruticola* and *M. viticola*, are also beginning to be of interest for enology.

## Figures and Tables

**Table 1:** Commercial products based on different *Metschnikowia* species, company, and recommended use.

Species	Products	Company	Applications
*M. pulcherrima*	Flavia™ MP346	Lallemand	In white wines, it increases the varietal thiols and improves the acidity
	Excellence™	Bio-Nature	To control the indigenous flora present in grape and in must it enables a controlled fermentation
*M. fructicola*	Gaïa™	Lallemand	To reduce the prefermentation sulfiting. To facilitate the implantation of the *S. cerevisiae* selected strain
	Noli™	Koppert	Tool for preventing the decay in fruit caused by *Botrytis* spp. and *Monilinia* spp*.,* which seriously affect the quality of harvested grapes

**Table 2:** Summary of final values of several wine quality parameters obtained in different studies that performed combined fermentations between *M. pulcherrima* and *S. cerevisiae* (Mp Sc) and control fermentations with *S. cerevisiae* (Sc).

Metabolite	Comitini et al., 2011 [19]		González-Royo et al., 2014 [25]		Benito et al., 2015 [26]		Chen et al., 2018 [27]		Escribano-Viana et al., 2018 [28]		Ruiz et al., 2018 [21]		Dutraive et al, 2019 [29]	
	Mp…Sc	Sc	Mp…Sc	Sc	Mp…Sc	Sc	Mp…Sc	Sc	Mp…Sc	Sc	Mp…Sc	Sc	Mp…Sc	Sc
Ethanol (% *v*/*v*)	13.87 ± 0.01	13.93 ± 0.06	10.6 ± 0.3	10.70 ± 0.10	13.61 ± 0.02	13.80 ± 0.01	12.60 ± 0.00	12.50 ± 0.06	14.15 ± 0.05	14.3	12.38 ± 0.38	13.00 ± 0.01	12.98 ± 0.35	13.20 ± 0.19
Residual sugar (g/L)	/	/	/	/	4.20 ± 0.17	4.65 ± 0.35	2.20 ± 0.30	2.30 ± 0.32	/	/	3.45 ± 0.36	3.27 ± 0.05	4.50 ± 0.30	4.40 ± 0.25
Lactic acid (g/L)	/	/	/	/	0.00 ± 0.00	0.00 ± 0.00	0.00 ± 0.00	0.00 ± 0.00	/	/	0.14 ± 0.03	0.10 ± 0.01	0.21 ± 0.01	0.21 ± 0.01
Malic acid (g/L)	/	/	/	/	/	/	1.7 ± 0.15	2.00 ± 0.11	1.87 ± 0.11	2.46	1.62 ± 0.06	1.83 ± 0.06	2.10 ± 0.01	2.28 ± 0.00
Acetic acid (g/L)	0.30 ± 0.04	0.46 ± 0.01	0.21 ± 0.02	0.18 ± 0.01	0.37 ± 0.02	0.38 ± 0.01	0.17 ± 0.05	0.19 ± 0.03	0.11 ± 0.01	0.16	0.36 ± 0.03	0.39 ± 0.01	0.30 ± 0.03	0.25 ± 0.03
Σ acids (g/L)	6.33 ± 0.27	7.05 ± 0.04	/	/	/	/	5.20 ± 0.10	5.60 ± 0.12	/	/	/	/	/	/
Glycerol (g/L)	6.53 ± 0.27	6.23 ± 0.54	5.3 ± 0.6	4.70 ± 0.01	6.12 ± 0.04	5.88 ± 0.02		//	8.27 ± 0.11	7.55			7.0 ± 0.05	5.8 ± 0.04
Acetaldehyde (mg/L)	41.84 ± 3.07	30.48 ± 1.97	1.57 ± 0.14	2.33 ± 1.11	33.00 ± 1.00	53.50 ± 2.12	7.94 ± 0.96	10.11 ± 1.20	/	/	39.00 ± 2.11	54.67 ± 5.13	/	/
2-phenyl-ethanol (mg/L)	9.49 ± 1.00	12.07 ± 0.403	/	/	19.10 ± 0.3	24.67 ± 1.44	27.82 ± 1.80	41.16 ± 27.25	64.99 ± 1.27	47.8	21.92 ± 0.09	17.81 ± 1.75	18.00 ± 0.60	13.74 ± 1.20
3-methyl-butanol (mg/L)	/	/	231.0 ± 23.8	178.60 ± 11.30	14.63 ± 0.74	16.54 ± 2.10	/	/	/	/	96.45 ± 5.34	163.85 ± 5.20	/	/
Hexanol (mg/L)	0.40 ± 0.08	0.38 ± 0.05	0.92 ± 0.05	0.92 ± 0.06	0.72 ± 0.16	1.05 ± 0.01	4.00 ± 0.09	3.74 ± 0.07	1.71 ± 0.21	1.82	0.60 ± 0.03	1.93 ± 0.75	1.28 ± 0.07	1.13 ± 0.04
Σ higher alcohols (mg/L)	290.23 ± 73.97	244.09 ± 42.51	311.7 ± 22.6	257.40 ± 16.80	161.43 ± 6.24	208.59 ± 17.83	/	/	153.43 ± 6.36	1.82	159.19 ± 10.00	236.55 ± 12.4	232.23 ± 11.84	203.48 ± 9.95
Ethyl butanoate (mg/L)	/	/	/	/	0.56 ± 0.13	558.92 ± 19.75	/	/	/	/	0.18 ± 0.01	0.15 ± 0.03	0.33 ± 0.02	0.43 ± 0.03
Ethyl hexanoate (mg/L)	0.14 ± 0.02	0.185 ± 0.02	0.68 ± 0.06	1.03 ± 0.22	1.33 ± 0.13	1.21 ± 0.03	/	/	/	/	0.57 ± 0.02	053 ± 0.01	1.99 ± 0.05	1.88 ± 0.07
Ethyl decanoate (mg/L)	0.15 ± 0.01	0.38 ± 0.01	0.14 ± 0.01	0.39 ± 0.11	0.68 ± 0.07	0.65 ± 0.02	/	/	/	/	0.27 ± 0.01	0.20 ± 0.02	0.55 ± 0.09	0.44 ± 0.06
Ethyl lactate (mg/L)	3.71 ± 0.73	7.66 ± 2.34	6.78 ± 0.23	8.01 ± 0.71	8.57 ± 0.19	9.76 ± 0.15	7.37 ± 1.19	10.06 ± 2.05	2.57 ± 0.11	0.48	/	/	/	/
Ethyl acetate (mg/L)	30.42 ± 2.18	28.44 ± 7.69	19.6 ± 0.4	24.40 ± 2.50	57.34 ± 3.72	75.48 ± 9.99	51.20 ± 12.78	31.50 ± 1.23	/	/	37.64 ± 1.36	0.11 ± 0.03	115.53 ± 6.80	159.18 ± 7.20
Isoamyl acetate (mg/L)	0.22 ± 0.01	0.26 ± 0.01	0.53 ± 0.02	0.46 ± 0.04	2.44 ± 0.10	3.67 ± 0.24	8.02 ± 3.35	6.61 ± 0.92	1.99 ± 0.30	2.13	1.15 ± 0.00	1.33 ± 0.25	/	/
2-phenyl-ethyl acetate (mg/L)	/	/	1.82 ± 0.20	2.37 ± 0.16	0.83 ± 0.06	0.96 ± 0.02	5.42 ± 0.40	6.60 ± 0.40	0.16 ± 0.01	0.13	0.34 ± 0.04	0.21 ± 0.03	0.36 ± 0.01	0.43 ± 0.09
Σ acetates (mg/L)	/	/	/	/	61.19 ± 3.6	80.73 ± 9.66	/	/	2.65 ± 0.40	2.26	/	/	120.29 ± 6.81	164.23 ± 7.36
Σ esters (mg/L)	/	/	/	/	73.88 ± 3.28	94.33 ± 8.93	/	/	/	/	41.26 ± 1.49	45.23 ± 11.23	124.84 ± 6.79	168.45 ± 7.37
Hexanoic acid (mg/L)	1.95 ± 0.07	0.80 ± 0.01	3.75±0.16	4.62 ± 0.35	12.82 ± 0.26	11.98 ± 0.97	/	/	/	/	7.09 ± 0.12	7.04 ± 0.19	11.38 ± 0.14	11.13 ± 0.30
Octanoic acid (mg/L)	1.02 ± 0.04	1.05 ± 0.01	15.86±0.70	20.11 ± 2.57	6.55 ± 0.09	6.72 ± 0.18	/	/	1.87 ± 0.23	2.57	5.55 ± 0.18	4.84 ± 0.35	11.56 ±0.75	11.99 ± 0.20
Decanoic acid (mg/L)	0.81 ± 0.04	0.23 ± 0.04	0.3±0.0	0.6 ± 0.00	2.92±0.13	3.1 ± 0.06	/	/	/	/	1.09 ± 0.06	0.68 ± 0.11	4.23±0.18	4.21 ± 0.14

**Table 3:** Variability among different strains of *M. pulcherrima* strains for reported phenotypic responses to physicochemical fermentation parameters.

Parameter	Du Plessis et al., 2017 [42] 7 strains of *M. pulcherrima*	Escribano et al., 2018 [55] 5 strains of *M. pulcherrima*	Canonico et al., 2019 [20] 7 strains of *M. pulcherrima*
Residual sugar (g/L)	2.63–0.00	/	/
Total acidity (g/L)	3.94–3.63	3.80–3.40	/
pH	3.70–3.51	/	/
Ethanol (% *v*/*v*)	9.58–8.10	/	4.04–3.50
Malic acid (g/L)	1.91–1.50	/	/
Volatile acidity (g/L)	0.37–0.21	0.16–0.05	0.37–0.26
Duration of AF (days)	46–180	/	/
